# Antibody Response to SARS-CoV-2 Vaccination in Patients with End-Stage Kidney Disease on Hemodialysis

**DOI:** 10.3390/vaccines11121802

**Published:** 2023-12-01

**Authors:** Rizky Andhika, Muhammad Iqbal Anand, Marita Restie Tiara, Josephine Debora, Hofiya Djauhari, Evan Susandi, Adnes Mareta, Asep Riswoko, Nopi Susilawati, Agnes Rengga Indrati, Bachti Alisjahbana, Rudi Supriyadi

**Affiliations:** 1 Division of Nephrology and Hypertension, Internal Medicine Department, Hasan Sadikin General Hospital, Faculty of Medicine, Universitas Padjadjaran, Bandung 40161, Indonesia; 2 Internal Medicine Department, Hasan Sadikin General Hospital, Faculty of Medicine, Universitas Padjadjaran, Bandung 40161, Indonesia; m.iqbal.anand@gmail.com (M.I.A.);; 3 Research Center for Care and Control of Infectious Diseases (RC3ID), Universitas Padjajaran, Bandung 40161, Indonesia; marita.restie@gmail.com (M.R.T.); hofiya@gmail.com (H.D.);; 4 Research Center for Polymer Technology—National Research and Innovation Agency (BRIN), Jakarta 10340, Indonesia; 5 Department of Clinical Pathology, Hasan Sadikin General Hospital, Faculty of Medicine, Universitas Padjadjaran, Bandung 40161, Indonesia; agnes.indrati@unpad.ac.id; 6 Division of Tropical and Infectious Diseases, Internal Medicine Department, Hasan Sadikin General Hospital, Faculty of Medicine, Universitas Padjadjaran, Bandung 40161, Indonesia

**Keywords:** COVID-19, chronic kidney disease, antibody level, vaccination

## Abstract

Patients with end-stage kidney disease on hemodialysis (ESKD-HD) have a high risk of contracting severe COVID-19. Vaccination can help reduce disease severity, but the immune dysregulation observed in these patients may result in an inadequate antibody response. Therefore, we aimed to evaluate the immune response postvaccination in ESKD-HD patients. This prospective cohort study was conducted in two hemodialysis centers in Indonesia. We enrolled ESKD-HD patients (*n* = 143) pre- and postvaccination and compared them to healthy subjects (*n* = 67). SARS-CoV-2 antibody response was assessed using anti-S-RBD antibodies and SVNT % inhibition tests. We performed bivariate and multivariate analysis to determine factors associated with SARS-CoV-2 antibody levels. Seropositive conversion was observed in 97% ESKD-HD subjects postvaccination. Compared with healthy subjects, ESKD-HD patients showed a comparable anti-S-RBD antibody titer postvaccination. mRNA vaccines remained a significant factor for the high immune response, while hypoalbuminemia correlated with lower immune response. In conclusion, ESKD-HD patients showed a robust immune response postvaccination. mRNA vaccines induced a stronger antibody response than other vaccines. Lower levels of serum albumin correlate with lower immune responses in ESKD-HD patients after vaccination.

## 1. Introduction

COVID-19 caused by the SARS-CoV-2 virus has shown a wide range of clinical manifestations, from asymptomatic to severe or critical illness. Patients with end-stage kidney disease (ESKD) in renal replacement therapy, including hemodialysis, are a vulnerable population with a higher mortality rate from COVID-19, ranging between 9% and 24% [1,2,3,4]. In addition, patients with end-stage kidney disease on hemodialysis therapy (ESKD-HD) are more likely to contract the disease because of the social interactions during hemodialysis sessions and frequent hospital visits [5]. Higher exposure and underlying immune dysregulation increase the probability of ESKD-HD patients acquiring severe COVID-19. A nationwide study in Qatar reported that 7.1% of all dialysis patients contracted COVID-19 [6]. In comparison, a study in Italy reported that COVID-19 incidence in nondialyzed CKD patients was 4.09% and 0.46% in the general population [7].

Vaccination is effective for lowering the infection incidence, severity, and mortality of COVID-19 among ESKD patients [8]. However, chronic immune dysregulation related to ESKD might affect the immune response after vaccination. As various factors are related to the lack of renal function and hemodialysis, ESKD patients may develop an inadequate antibody response when compared to the general population [9,10]. A meta-analysis reported that ESKD patients undergoing hemodialysis, in comparison to a normal population, showed lower seroconversion rates and level of seroprotection after vaccination against viral respiratory disease, for both influenza (H1N1 and H3N2) and COVID vaccination [11]. However, another vaccine study reported that the majority of patients on hemodialysis displayed an adequate humoral response following full-dose vaccination with the BNT162b2 vaccine. The median antibody level, though, was significantly lower than that of the healthy control group (2900 vs. 7401, *p* < 0.001, respectively) [9]. In addition, ESKD-HD patients also displayed an earlier decline in anti-SARS-CoV-2 antibody titers [12].

The development of an immune response in ESKD patients is associated with several factors. Factors related to a poor serological response include the use of immunosuppressive drugs, poor nutritional status, lower lymphocyte count, lower hemoglobin and albumin levels, longer dialysis duration, and high intravenous iron dose [9,10,13]. Older age has also been reported to be associated with a lower immune response [9].

Limited studies are available that have evaluated the immune response in ESKD patients postvaccination. Therefore, we aimed to determine the immune response following COVID-19 vaccination with CoronaVac (Sinovac Life Sciences, Beijing, China), mRNA-1273 (Moderna Inc., Cambridge, MA, USA), and BNT162b2 (Pfizer/BioNTech, Mainz, Germany) in patients with ESKD-HD, and analyzed the factors that may affect the humoral response.

## 2. Materials and Methods

### 2.1. Study Design and Participants

We conducted a prospective cohort study in hemodialysis centers in Hasan Sadikin General Hospital, Bandung, and Slamet General Hospital, Garut, West Java, Indonesia, from September to November 2021. We enrolled ESKD-HD patients from the respective centers. For comparison, healthy control subjects were enrolled in a research clinic in the Medical Faculty of Padjadjaran University, Bandung.

**Figure 1:**
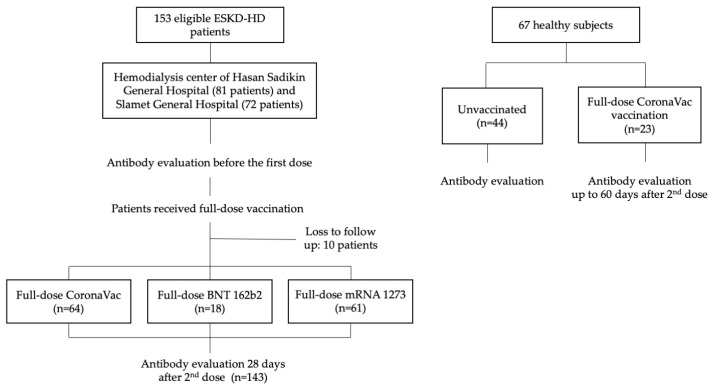
Flowchart of patient enrollment.

First, we enrolled patients in September and October 2021 to acquire prevaccination data, and these subjects were reassessed in November 2021 for antibody levels after receiving full-dose vaccination. In this study, we used convenience sampling, wherein we invited ESKD-HD patients who were willing to participate in the study. Inclusion criteria were age ≥ 18 years, listed on a routine hemodialysis program, and intention to receive CoronaVac (Sinovac Life Sciences, Beijing, China), mRNA-1273 (Moderna Inc., Cambridge, MA, USA), or BNT162b2 (Pfizer/BioNTech, Mainz, Germany) vaccines in their respective institutions following their enrollment. All HD patients in both dialysis centers underwent hemodialysis twice weekly. The exclusion criteria were those unwilling to participate in the study, or previously vaccinated patients. The flowchart of patient selection and subsequent analysis is presented in Figure 1.

In Hasan Sadikin Hospital, we enrolled 74 patients, of whom 56 (75.7%) received Coronavac, and 18 (24.3%) received BNT162b2 vaccine. In the Slamet General Hospital, we enrolled 69 patients, of whom 8 (11.6%) received CoronaVac, and 61 (88.4%) received mRNA-1273 vaccine. Healthy control subjects were enrolled earlier in the year 2021. First sampling of unvaccinated subjects was conducted in June 2021, while antibody examination after full-dose vaccination was conducted between July and December 2021. The antibody response examination among healthy control subjects was not paired. This meant that the person being examined before vaccination was different than the subject tested after vaccination (Figure 1).

### 2.2. Data Collection and Outcome

After obtaining informed consent from the subjects, we collected baseline information on factors that may affect the immune response to vaccination by using questionaries. Data collected included clinical characteristics such as age, sex, body mass index (BMI), presumed etiology of ESKD, and the duration of hemodialysis (months). We collected 5 mL blood from all patients for measurement of anti-SARS-CoV-2-SRBD antibody (anti-S-RBD) and surrogate viral neutralization test (SVNT). These tests were conducted at the beginning of the dialysis session for ESKD-HD patients. Patients were also tested for their hematology parameters (hemoglobin, leucocyte count) and plasma albumin level.

Body mass index was defined as dry weight (in kg) divided by height (in square meters). Duration of hemodialysis was classified into two groups: <5 years and ≥5 years. Hemoglobin level was classified into two groups: <8 g/dL and ≥8 gr/dL, according to the WHO cut-off of severe anemia [14]. Leukocyte count was classified into two groups according to the leucopenia cut-off: <4300 cells/µL and ≥4300 cells/µL. Finally, albumin levels were also classified as <3.4 g/dL and ≥3.4 g/dL.

### 2.3. Measurement

We assessed antibodies using the FastBioRBD™ (Wondfo, Guangzhou Biotech, Guangzhou, China) anti-S-RBD antibody tests and the GenScript c-Pass SARS-CoV-2 Surrogate Viral Neutralization Detection Kit (GenScript c-Pass Biotech, Leiden, The Netherlands). For distribution in Indonesia, FastBioRBD™ was rebranded by PT Biofarma (Persero), Bandung, Indonesia. FastBioRBD™ quantifies the anti-S-RBD antibody in serum with a phosphorescent marker using a fluorescence-based immunoassay (FIA). The test result is given in arbitrary unit (AU)/mL. The results are confirmed as seropositive if ≥1 AU/mL, with a maximum detection limit of 200 AU/mL [15].

The SARS-CoV-2 SVNT was carried out according to the manufacturer’s instruction [16], and the percentage of inhibition was calculated as follows: inhibition [%] = (1 − (sample OD450/average negative control OD450)) × 100. The cut-off value for positive presence of neutralizing antibodies was at 30% inhibition. The FastBioRBD™ compared to the GenScript c-Pass had a good correlation (r-value: 0.86, 95% confidence interval [CI]: 0.83–0.89, *p* < 0.0001) (Figure S1). Samples with discrepant results between the tests were retested for both SVNT and anti-S-RBD antibody, and the best results were taken.

### 2.4. Statistical Analysis

The clinical characteristics of the participants were presented according to the data types. Categorical data were presented as frequencies and percentages. Meanwhile, for continuous data, we initially conducted a normality test using the Shapiro–Wilk test. If the data followed a normal distribution, we presented them using means and standard deviations (SD); if not, we presented them using medians and interquartile ranges (IQRs). Bivariate and multivariate analyses were performed to determine the relationship between each factor and the level of SARS-CoV-2 antibodies. Since the SARS-CoV-2 antibody levels were not normally distributed, we used the Mann–Whitney test to analyze the antibody levels between the two groups, and the Wilcoxon test for the pre- and postgroups. Furthermore, to assess differences among more than two groups, we applied the Kruskal–Wallis test, followed by post hoc analysis using Dunn’s test.

We further conducted bivariate and multivariate analyses to test factors related to having a high antibody response. Owing to the non-normal data distribution, we use binomial categories of high versus low anti-S-RBD and SVNT level. For the prevaccination assessment, we set the anti-S-RBD level of ≥59.76 AU/mL and SVNT 60% inhibition level as the high antibody level cut-off [17]. In the postvaccination assessment, high antibody level was determined if the maximum (200 AU/mL) level of anti-S-RBD or >90% inhibition level of SVNT was reached. A multivariate logistic regression analysis was also carried out.

All statistical tests were two-sided, and a *p* value of <0.05 was considered to indicate statistically significant differences. All data were analyzed using Statistical Product and Service Solution (SPSS) version 25.0 for Windows (IBM Corporation, Armonk, NY, USA) and GraphPad PRISM (version 9).

### 2.5. Ethical Considerations

This study was reviewed and approved by the institutional review board/ethics committee of the Hasan Sadikin General Hospital and Faculty of Medicine, Universitas Padjadjaran (ethics approval No. 410/UN6.KEP/EC/2021). This study was conducted according to the tenets of the Declaration of Helsinki and Good Clinical Practices. All participants were informed of the nature of the study, and provided their consent for participation. No personal data of participants are disclosed in this study.

## 3. Results

### 3.1. Study Sample

We enrolled 143 subjects with ESKD on a routine hemodialysis schedule, from September to December 2021. A control group of 67 healthy adults was also selected; of these, 44 subjects were unvaccinated and 23 were examined after full-dose vaccination. Baseline characteristics of the subjects are presented in Table 1. All healthy subjects received the CoronaVac vaccination. Meanwhile, three types of vaccines were administered to the ESKD-HD group, namely, CoronaVac, mRNA-1273, and BNT162b2 vaccines, with 64, 61, and 18 recipients, respectively. A similar proportion of female and male patients was included in this study (50.4 vs. 49.6%). The median age of ESKD patients was higher than that of the healthy subjects (median age of 48 (40–55) vs. 31 (25–42), respectively).

The median value of hemoglobin in all subjects was 8.6 (7.8–9.6) g/dL, and in all groups, the median value of hemoglobin was higher than 8. Severe anemia, or hemoglobin <8 g/dL, was observed in 27.9% subjects. The median leucocyte count was 5930 (4490–7385) cells/L, with all groups exhibiting a median of >4300 cells/L. Additionally, the median albumin level in all participants was 3.6 (3.2–4) g/dL, and hypoalbuminemia was observed in 32.9% subjects. A median albumin of <3.4 g/dL was observed in subjects receiving CoronaVac and BNT162b2, while those receiving mRNA-1273 had a higher level of albumin (median: 4.0 (3.8–4.3) g/dL).

The median duration of hemodialysis in all subjects was 50 (28–81) months. Lower median duration was observed in subjects receiving the mRNA-1273 vaccine, and higher in subjects receiving the BNT162b2 vaccine, with medians of 40 (20–60) and 62 (31–78) months, respectively. Hypertension was found to be the main etiology of ESKD in all groups, with percentages of 60.9%, 77.0%, and 77.7% in the CoronaVac, mRNA-1273, and BNT162b2 vaccine groups, respectively.

**Figure 2:**
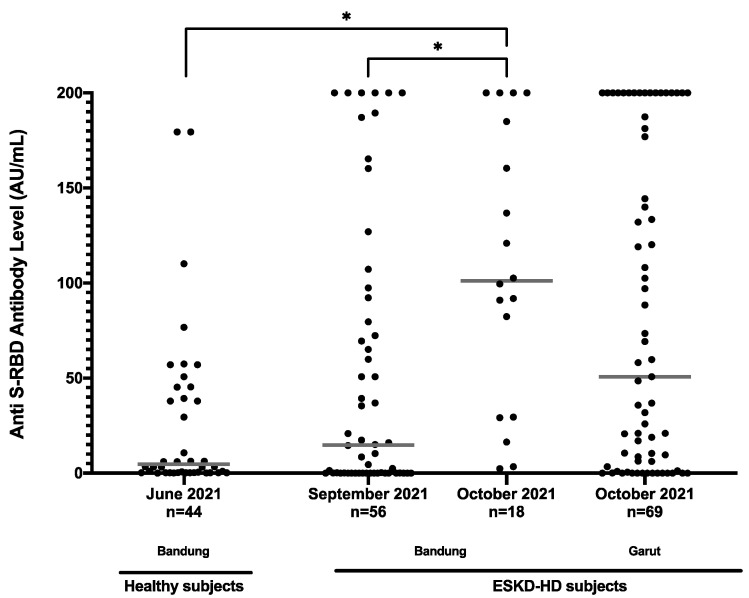
Anti-S-RBD antibody level (AU/mL) of all prevaccination subjects based on time collected. * *p* < 0.05.

We observed a higher median level of prevaccination anti-S-RBD in subjects enrolled later than earlier. Healthy subjects who were enrolled earlier also showed the lowest median value of anti-S-RBD antibody level of 4.7 (IQR: 0.4–49.4) AU/mL. The first batch of sampling of ESKD-HD subjects was conducted in September and October 2021, and the second in November 2021. Anti-S-RBD antibody level in patients enrolled later showed a significantly higher median value of 101.1 (IQR: 29.5–188.7) AU/mL versus 14.8 (IQR: 0.08–89.1) AU/mL. Healthy control subjects were enrolled in the same city as the ESKD-HD patients in Hasan Sadikin General Hospital, but Slamet Hospital is in a different city (Figure 2).

### 3.2. Anti-S-RBD Antibody Level in ESKD Patients before and after Vaccination

**Figure 3:**
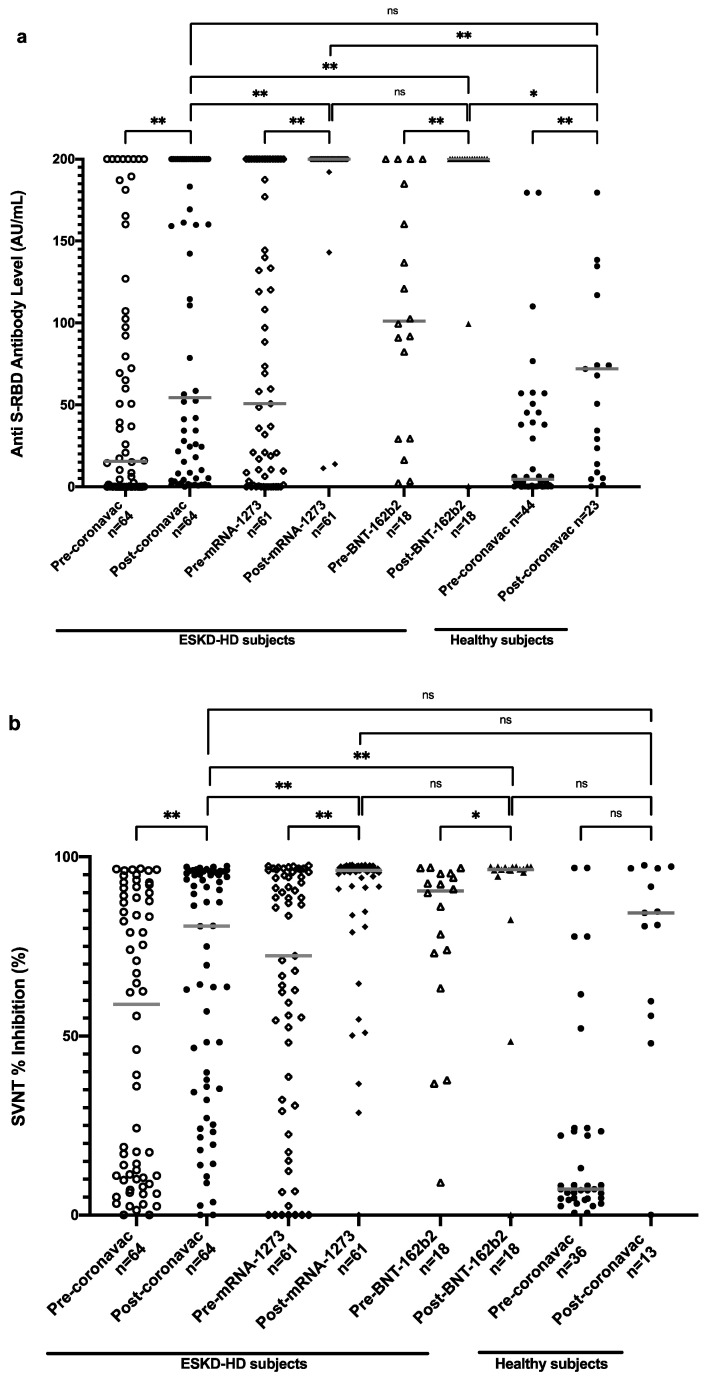
Anti-S-RBD antibody level (**a**) and SVNT % inhibition (**b**) in all subjects pre- and postvaccination based on vaccine types. Grey lines depict the median; ns: nonsignificant; * *p* < 0.05; ** *p* < 0.001.

Figure 3a shows the distribution of anti-S-RBD antibody titers in all subjects. An increasing trend of anti-S-RBD antibody levels in all types of vaccines was obtained after one month of full-dose vaccination. ESKD-HD subjects vaccinated with CoronaVac had a median value of anti-S-RBD antibody of 15.59 AU/mL before vaccination, which rose to 54.48 AU/mL one month after complete vaccination. Those vaccinated with mRNA-1273 had a median anti-S-RBD antibody value of 50.72 AU/mL prevaccination, which rose to 200 AU/mL one month after complete vaccination. Subjects vaccinated with BNT162b2 had a median anti-S-RBD antibody value of 101.11 AU/mL, which rose to 200 AU/mL one month after complete vaccination. Hence, significantly higher levels of antibodies were obtained in patients vaccinated with mRNA-1273 and BNT162b2 than with CoronaVac. When compared with healthy subjects, ESKD-HD patients showed a comparable median value of anti-S-RBD antibody post-CoronaVac vaccination.

### 3.3. Neutralization Test in ESKD Patients before and after Vaccination

The distribution of the neutralization test results of all subjects is shown in Figure 3b. An increasing trend of SVNT % inhibition values was detected in subjects after vaccination with CoronaVac, mRNA-1273, and BNT162b2. Subjects vaccinated with CoronaVac had an increase in SVNT % inhibition from 58.91% before vaccination to 80.69% one month after complete vaccination. Subjects vaccinated with mRNA-1273 had a median value of 72.35% inhibition prevaccination, which rose to 96.24% one month after complete vaccination. Subjects vaccinated with BNT162b2 had a higher median value of SVNT % inhibition of 90.50% before vaccination, which increased to 96.45% one month after full vaccination. Similar to anti-S-RBD titers, ESKD-HD patients showed comparable SVNT % inhibition compared to healthy subjects. A significantly higher increase of SVNT % inhibition was also observed in patients vaccinated with mRNA-1273 and BNT162b2 vaccines than with CoronaVac.

### 3.4. Factors Influencing Anti-S-RBD Antibody Level and SVNT % Inhibition Prevaccination

In the combined analysis of all vaccine types, we observed that sex and history of COVID-19 infection affected antibody levels before vaccination (Table 2). Female patients exhibited higher prevaccination antibody levels than male patients. Likewise, subjects with a history of COVID-19 infection also had higher levels of antibodies before vaccination than those who have never been infected before.

Higher anti-S-RBD levels and % inhibition were found in patients with a longer duration of hemodialysis and lower leucocyte count, but these differences were not statistically significant. Subjects with lower hemoglobin and lower albumin levels showed higher levels of anti-S-RBD and % inhibition. However, the association was not significant (Table 2).

In the multivariate analysis, we confirmed that higher anti-S-RBD prevaccination correlates strongly with history of COVID-19 (Table S2a). For SVNT, it also correlated with lower leucocyte count (Table S2b).

### 3.5. Factors Influencing Anti-S-RBD Antibody Level and SVNT % Inhibition Postvaccination

While sex and history of COVID-19 were significantly associated with higher anti-S-RBD antibody and SVNT levels in the prevaccinated condition, this association was not seen postvaccination. However, the effect of history of COVID-19 remains associated with antibody level. In addition, we observed that having glomerulonephritis and lower albumin is associated with a lower antibody response (Table 2). The highest factor associated with higher anti-S-RBD and SVNT % inhibition was full-dose vaccination. CoronaVac showed a modest response, while BNT162b2 and mRNA-1273 showed a very high response (Figure 3).

Other factors such as age, sex, and longer duration of hemodialysis were not associated with a lower antibody response. Moreover, laboratory parameters such as lower hemoglobin and leucocyte level were not associated with lower antibody response. Lower albumin level, on the other hand, showed significantly lower anti-S-RBD and SVNT % inhibition levels.

**Figure 4:**
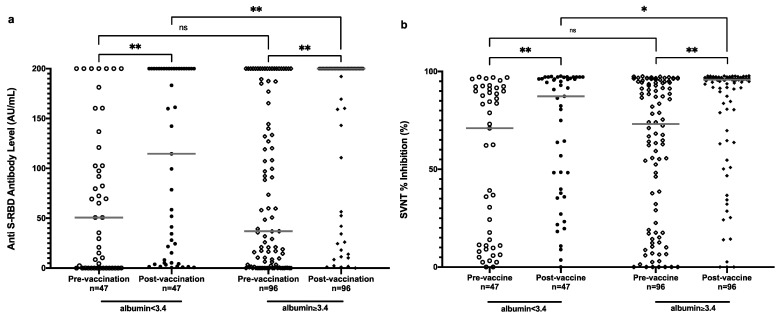
Anti-S-RBD antibody level (**a**) and SVNT % inhibition (**b**) in all subjects pre- and postvaccination based on albumin level. Grey lines depict the median; ns: nonsignificant; * *p* < 0.05; ** *p* < 0.001.

It is interesting that patients with lower hemoglobin and albumin showed a higher antibody level at the prevaccination level. However, the effect remained for hemoglobin levels, but reversed for albumin levels postvaccination. Postvaccination, lower albumin levels showed significant association with lower anti-S-RBD antibody levels, as well as SVNT % inhibition (Table 2 and Figure 4).

After categorizing patients into the high and low responders groups postvaccination, we performed bivariate and multivariate analysis of factors associated with high responders. Observing the anti-S-RBD result, we found that vaccine type was the most important factor that was significantly associated with higher antibody levels for the mRNA vaccine and BNT vaccine (adjusted odds ratio [OR]: 19.1, 95%CI: 4.9–73.9 and adjusted OR: 9.2, 95%CI: 0.8–104.6, respectively) (Table 3). A history of having COVID-19 still showed significant association with having a higher antibody response. However, a significant effect of sex was not observed in the postvaccinated status. Similar results were also shown in the SVNT % inhibition level (Table 4).

## 4. Discussion

In this study, we found that vaccination of ESKD-HD subjects resulted in an adequate increase of anti-S-RBD antibody level and SVNT % inhibition, regardless of the vaccine type. Of all the ESKD-HD subjects, 97% developed a seropositive conversion after full-dose vaccination. This finding is similar to previous studies that reported that an adequate seroconversion could be seen in most ESKD-HD patients [8,9,13]. The lower antibody response compared to non-ESKD patient has been previously reported by others [9,10,13,18], but our findings showed that the antibody response in the ESKD-HD patients were not inferior to the healthy subjects.

We found a modest increase of immune response in ESKD subjects following CoronaVac vaccination. Vaccinations with mRNA-1273 and BNT162b2 resulted in significantly higher anti-S-RBD antibody level than with CoronaVac. Similarly, the increasing pattern and significance were also shown in the % inhibition tested by SVNT in all vaccine types. This result is in accordance with a Chilean study that reported that the BNT162b2 vaccine induced a significantly higher humoral response than the CoronaVac vaccine [19]. Previous studies in Indonesia and Turkey reported seropositivity of only 86% and 86.7% at 1 and 3 months after receipt of two doses of inactivated COVID-19 vaccination [20,21], respectively.

A lower humoral immune response induced by inactivated vaccines might be related to the difference in cellular immunity elicited by these vaccines compared to mRNA vaccines. One study evaluated a detailed characterization of cellular immunity elicited by inactivated virus compared with mRNA vaccines in a healthy adult population. The study reported the inactivated vaccines induced broader T cell immunity, but lower quantity of spike peptide-stimulated T cell cytokines compared with mRNA vaccines. The T cell response of inactivated vaccines was also mainly mediated only by CD4 T cells, not a coordinated CD4 and CD8 T cell expansion [22]. Meanwhile, when comparing the mRNA vaccines, previous studies reported higher seroconversion in mRNA-1273 compared with BNT162b2 vaccines [13,18]. The hypothesized explanation was that the mRNA-1273 vaccine comprised a higher mRNA dose (100 μg) than the BNT162b2 vaccine (30 μg) [18].

We first conducted the study in September and October 2021. It was interesting to note that even with a difference of only 1 month between patient inclusion, a significant increase of the baseline antibody level could be seen. Higher baseline antibodies were observed in subjects that were enrolled later. We hypothesized that natural infection played a part in this finding, since the inclusion was during the SARS-CoV-2 Delta variant outbreak. We believe that regular visits by ESKD-HD patients to the hospital facilitated high exposure to COVID-19. The high incidence of COVID-19 in patients with regular dialysis has been previously reported, ranging between 7.1 and 11.9% [6,18].

We also found that a lower albumin level was a factor affecting the lower immune response after vaccinations. Consistent with our findings, low albumin has been linked with a lower antibody response after vaccination [13,18,23]. The detailed mechanisms of how albumin affects the immune response following vaccination have not been completely elucidated. Albumin is known to have anti-inflammatory and antioxidant properties, and binds to various inflammatory mediators, thereby showing involvement in the regulation of immune response [24,25]. However, perhaps more importantly, a lower albumin level indicates that the nutrition level is low [26]. Other factors that affected the immune response were history of COVID-19, vaccine types, immunosuppressive drug treatment, lymphocyte count, and duration of dialysis [13,18].

It is intriguing that higher antibody levels were found in the group with lower hemoglobin levels at prevaccination assessment. However, after a full dose of vaccination, this difference evened off. Other studies found that hemoglobin positively correlated with antibody titer after COVID-19 vaccination [13,27,28]. Low hemoglobin in ESKD patients is associated with erythropoietin resistance, which is known to be a state predominantly dependent on inflammation, and chronic inflammation has been linked with a poorer humoral immune response. Therefore, low hemoglobin correlates with lower immune response postvaccination [28]. After a stratified analysis, we found that the high antibody levels among prevaccinated subjects with low hemoglobin were caused by the higher frequency of having a history of COVID-19.

In this study, we used the FIA antibody test for determination of the anti-S-RBD antibody level. Validation of the anti-S-RBD antibody test showed good correlation with SVNT. SVNT is considered a more sensitive assay than anti-S-RBD, but for general or regular use, this anti-S-RBD antibody test is acceptable and shows good sensitivity, specificity, and agreement values [29]. For the screening tests, we opted to use an assay with higher specificity to ensure that high antibody levels are most likely to be associated with high neutralization levels. Since the anti-S-RBD antibody test that we used is a point-of-care test, it can easily be deployed in any setting, and may support vaccination programs, even in a field setting [30].

Our study was limited by the small sample size, which may not be sensitive enough to show the subtle differences of antibody responses and the affecting factors among the groups. Time differences in conducting the measurement of prevaccine antibody levels also confounded the results. We conducted sampling for CoronaVac first, followed by mRNA-1273 and BNT162b2. Prevaccination sampling of healthy subjects was performed even earlier in July 2021, just before the Delta variant outbreak. Subsequent prevaccination enrollment was associated with higher levels of anti-S-RBD antibody and SVNT % inhibition, which subsequently affected the postvaccination results. Our study only assessed the humoral immunity of vaccine responses, and not cellular immunity. Last, due to technical constraints, we did not use the plaque-reduction neutralization test to assess antibody response.

## 5. Conclusions

Patients with ESKD are prone to transmission of and having severe infection from COVID-19. All three tested vaccines showed a favorable increase in antibody response and neutralization capacity. The mRNA vaccines induced a stronger antibody response than inactivated viral vaccines. Low levels of serum albumin were associated with a lower antibody response in ESKD-HD patients after vaccination. Based on these findings, we report that administration of the COVID-19 vaccine will produce a good response in most patients with ESKD. However, if available, the mRNA vaccine is preferred over inactivated vaccine. Additional tests to determine the levels of anti-S-RBD antibody achieved after vaccination can help us to ensure that the patient has acquired adequate immunoprotection.

## Figures and Tables

**Table 1:** Baseline characteristics of subjects.

Characteristics	ESKD-HD Subjects			Healthy Subjects (n = 67) CoronaVac
	CoronaVac (n = 64)	mRNA-1273 (n = 61)	BNT162b2 (n = 18)	
Age (years), median (IQR)	46 (38–55)	47 (40–55)	52 (47–59)	31 (25–42)
Age categories, n (%)				
18–29 years	6 (9.4)	4 (6.6)	0 (0)	26 (39.4)
30–39 years	10 (15.6)	11 (18.0)	2 (11.1)	17 (25.8)
40–49 years	19 (29.7)	21 (34.4)	6 (33.3)	18 (27.3)
50–59 years	21 (32.8)	15 (24.6)	6 (33.3)	1 (1.5)
≥60 years	8 (12.5)	10 (16.4)	4 (22.2)	4 (6.1)
Sex, n (%)				
Male	37 (57.8)	25 (41.0)	3 (16.7)	39 (58.2)
Female	27 (42.2)	36 (59.0)	15 (83.3)	28 (41.8)
Laboratory findings, median (IQR)				
Hb (g/dL)	8.6 (7.8–9.8)	8.4 (7.8–9.2)	9.0 (8.1–9.6)	—
Leukocyte (cells/µL)	5760 (4457–7270)	6340 (4560–7520)	5680 (4448–7322)	—
Albumin (g/dL)	3.3 (2.9–3.6)	4.0 (3.8–4.3)	3.23 (2.87–3.63)	—
Body mass index (kg/m^2^), median (IQR)	23.0 (20.9–24.3)	23.1 (20.2–25.2)	23.5 (21.3–28.2)	23.4 (20.5–26.8)
Duration of HD (months), median (IQR)	50 (28–103)	40 (20–60)	62 (31–78)	—
Etiology of ESKD, n (%)				
Diabetic Kidney Disease	11 (17.2)	2 (3.3)	1 (5.6)	—
Glomerulonephritis	11 (17.2)	0 (0)	2 (11.1)	—
Hypertension	39 (60.9)	47 (77.0)	14 (77.7)	—
Others	3 (4.7)	12 (19.7)	1 (5.6)	—
History of COVID-19				
Yes	1 (1.6)	16 (26.2)	15 (83.3)	17 (25.4)
No	63 (98.4)	45 (73.8)	3 (16.7)	50 (74.6)
Anti-S-RBD antibody level (AU/mL), median (IQR)				
Prevaccination	15.6 (0.1–101.2)	50.7 (2.3–193.7)	101.1 (29.5–188.7)	4.7 (0.3–49.4)
1 month after complete vaccination	54.5 (6.0–200)	200 (200–200)	200 (200–200)	72.0 (13.9–179.7)
SVNT (% inhibition), median (IQR)				
Prevaccination	58.9 (10.1–89.0)	72.35 (29.8–94.6)	90.50 (70.7–95.4)	7.2 (4.6–23.4)
1 month after complete vaccination	80.7 (32.7–95.3)	96.24 (93.0–97.1)	96.45 (95.4–97.2)	84.4 (57.7–97.7)

**Table 2:** Anti-S-RBD antibody and SVNT % inhibition levels in all ESKD-HD subjects.

Characteristics	Total (n = 143)	Anti-S-RBD Antibody Level (AU/mL) Median (IQR)		SVNT (% Inhibition) Median (IQR)	
		Prevaccination	Postvaccination	Prevaccination	Postvaccination
Age categories					
18–29 years	10	9.5 (0.0–29.4)	180 (52.3–200)	33.3 (0.0–68.4)	93.9 (76.6–96.3)
30–39 years	23	26.0 (0.1–107.3)	200 (34.3–200)	59.4 (22.6–92.2)	96.4 (56.9–97.3)
40–49 years	46	54.5 (5.5–190.6)	200 (102.7– 200)	83.6 (28.6–94.8)	95.9 (80.3–96.5)
50–59 years	42	33.2 (0.1–102.5)	200 (22.2–200)	63.3 (11.2–91.1)	94.9 (46.2–96.5)
≥60 years	22	100.3 (15.8–186.0)	200 (54.4–200)	88.6 (46.2–93.5)	95.4 (62.1–97.7)
Sex					
Male	65	10.5 (0.9–99.9) *	200 (49.2–200)	55.8 (10.1–88.5)	95.6 (63.8–97.7)
Female	78	66.8 (8.9–188.0)	200 (72.1–200)	84.8 (43.9–94.3)	95.3 (64.1–96.6)
Etiology of ESKD					
Diabetic Kidney Disease	14	8.2 (0.1–125.7)	107.8 (12.8–200)	40.6 (5.3–89.5)	83.6 (34.8–95.9)
Glomerulonephritis	13	15.1 (0.1–80.7)	51.9 (5.5–137.2) *	36.8 (11.1–91.9)	64.4 (40.9–94.0) *
Hypertension	100	58.9 (4.9–174.1)	200 (172.8–200)	82.7 (33.2–93.8)	95.7 (68.2–96.7)
Others	16	23.4 (0.1–172.8)	200 (200–200)	43.1 (0.0–92.1)	96.2 (95.6–96.5)
History of COVID-19 infection					
Yes	32	101.1 (29.3–200) *	200 (200–200) *	90.5 (72.6–96.3)	96.4 (95.7–97.2) *
No	111	18.9 (0.1–127)	200 (26.0–200)	59.4 (10.4–91.3)	94.2 (50.2–96.4)
Duration of hemodialysis (years)					
<5	89	31.8 (0.91–152.4)	200 (151.1–200)	68.2 (17.5–68.2)	95.9 (64.1–96.8)
≥5	54	62.4 (0.09–161.5)	200 (32.8–200)	78.6 (12.1–92.3)	93.98 (62.0–96.8)
Hemoglobin (g/dL)					
<8	40	54.4 (7.2–194.2)	200 (70.9–200)	90.2 (27.2–96.4) *	96.3 (67.1–97.2)
≥8	103	29.2 (0.0–139.9)	200 (52.5–200)	64.1 (12.6–90.1)	94.6 (63.0–96.4)
Leukocyte (cells/µL)					
<4300	31	15.11 (0.1–121.0)	200 (26.0–200)	54.4 (11.3–86.6)	91.4 (46.7–96.8)
≥4300	112	49.6 (1.0–164.1)	200 (111.7–200)	76.92 (17.21–93.37)	95.8 (76.4–96.6)
Albumin (g/dL)					
<3.4	47	50.71 (0.1–120.9)	114.6 (5.3–200) *	71.0 (11.0–91.0)	87.3 (37.8–96.5) *
≥3.4	96	36.9 (1.28–183)	200 (200–200)	73.2 (17.9–94.3)	95.8 (83.9–96.6)

Note: significant difference between group categories at * *p* < 0.05.

**Table 3:** Bivariate and multivariate analysis of factors associated with high anti-s-rbd antibody level (≥200 AU/mL) postvaccination.

Variables	Bivariate Analysis		Multivariate Analysis	
	Odds Ratio (OR)	*p*-Value	aOR	*p*-Value
Sex (female)	1.2 (0.6–2.3)	0.654	0.4 (0.1–1.1)	0.079
Duration of HD (≥5 years)	0.7 (0.3–1.3)	0.259	1.0 (0.4–2.9)	0.970
History of COVID-19 (yes)	24.5 (3.2–185.8)	0.000 *	12.6 (0.9–180.7)	0.062
Vaccine types (mRNA-1273)	31.4 (9.9–98.3)	0.000 *	19.1 (4.9–73.9)	0.000 *
Vaccine types (BNT162b2)	17.6 (3.7–83.9)	0.000 *	9.2 (0.8–104.6)	0.074
Hemoglobin (<8 g/dL)	1.4 (0.6–2.9)	0.438	2.1 (0.7–6.7)	0.209
Leucocyte (<4300 cells/µL)	0.5 (0.2–1.1)	0.077	0.3 (0.1–0.9)	0.034 *
Albumin (<3.4 g/dL)	0.2 (0.2–0.4)	0.000 *	0.3 (0.1–0.9)	0.026 *

Dependent variable: anti-S-RBD antibody level ≥ 200 AU/mL; *: statistically significant.

**Table 4:** Bivariate and multivariate analysis of factors associated with high (≥90%) surrogate viral neutralization test (% inhibition) postvaccination.

Variables	Bivariate Analysis		Multivariate Analysis	
	Odds Ratio (OR)	*p*-Value	aOR	*p*-Value
Sex (female)	0.9 (0.5–1.9)	0.949	0.5 (0.2–1.2)	0.125
Duration of HD (≥5 years)	0.5 (0.2–0.9)	0.039 *	0.5 (0.2–1.2)	0.158
History of COVID-19 (yes)	2.9 (1.1–7.8)	0.023 *	1.0 (0.3–3.8)	0.946
Vaccine types (mRNA-1273)	6.2 (2.7–14.1)	0.000 *	4.5 (1.6–12.8)	0.004 *
Vaccine types (BNT162b2)	6.8 (1.8–26.0)	0.005 *	11.9 (1.9–71.5)	0.007 *
Hemoglobin (<8 g/dL)	1.7 (0.8–3.7)	0.204	1.9 (0.8–5.1)	0.164
Leucocyte (<4300 cells/µL)	0.6 (0.3–1.3)	0.212	0.5 (0.2–1.2)	0.134
Albumin (<3.4 g/dL)	0.4 (0.2–0.8)	0.007 *	0.5 (0.2–1.2)	0.144

Dependent variable: SVNT % inhibition ≥ 90%; *: statistically significant.

## Data Availability

The data used to support the findings of this study were included in the article.

## Supplementary Materials

The following supporting information can be downloaded at: https://www.mdpi.com/article/10.3390/vaccines11121802/s1, Figure S1: Linear Correlation between anti-S-RBD antibody level and SVNT % Inhibition.; Figure S2: Ethical approval from the institutional review board; Figure S3: Informed consent form. Table S1a: Anti-S-RBD Antibody Level (AU/mL) and SVNT (% inhibition) in ESKD Subjects Vaccinated with CoronaVac; Table S1b: Anti-S-RBD Antibody Level (AU/mL) and SVNT (% inhibition) in ESKD Subjects Vaccinated with mRNA-1273; Table S1c: Anti-S-RBD Antibody Level (AU/mL) and SVNT (% inhibition) in ESKD Subjects Vaccinated with BNT162b2; Table S2a: Bivariate and multivariate analysis of factors associated with high anti-S-RBD Antibody Level (≥59.76 AU/mL) prevaccination; Table S2b: Bivariate and multivariate analysis of factors associated with high SVNT % Inhibition (≥60%) prevaccination.
