# Supplementary material for: Antibody Response to SARS-CoV-2 Vaccination in Patients with End-Stage Kidney Disease on Hemodialysis
Source: Vaccines (Basel). 2023 Dec 1;11(12):1802. doi: 10.3390/vaccines11121802 (PMC10747993; doi:10.3390/vaccines11121802)
Supplement: Supplementary file 1 [file vaccines-11-01802-s001.zip › vaccines-2633745-supplementary.pdf]

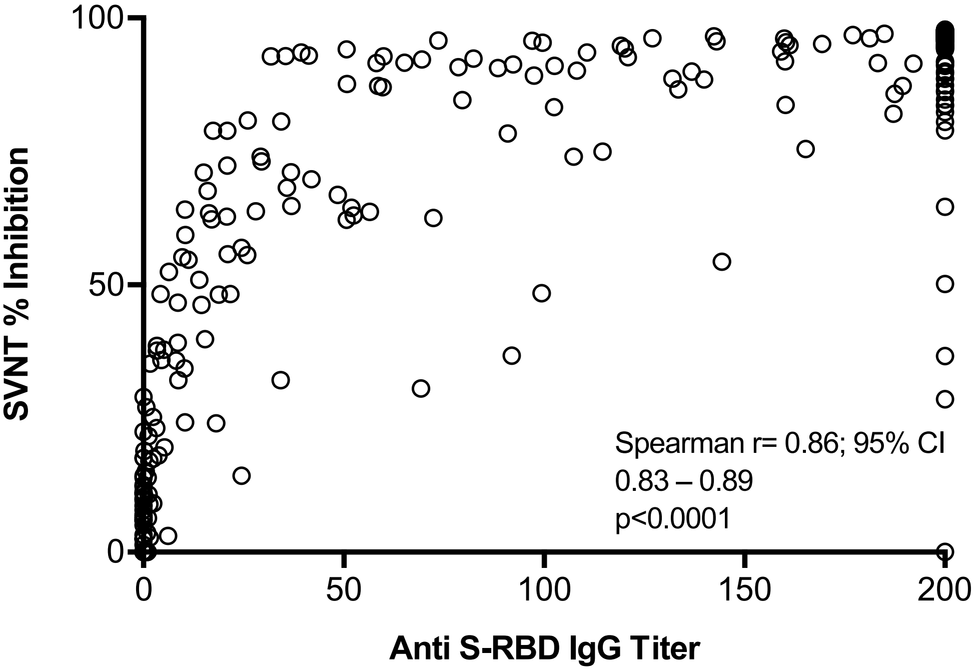

Figure S1. Linear Corelation between anti S-RBD antibody level and SVNT % Inhibition.

Table S1a. Anti-S-RBD Antibody Level and SVNT (% Inhibition) in ESKD Subjects Vaccinated with CoronaVac.

Variables	n	Anti S-RBD Antibody Level (AU/mL), median (IQR)				SVNT (% inhibition), median (IQR)			
		pre-vaccination	p-value	post-vaccination	p-value	pre-vaccination	p-value	post-vaccination	p-value
**Duration of HD (years)**									
<5	36	14.8 (0.1—91.2)	0.997	107.8 (4.5—200)	0.536	50.9 (11.8—86.3)	0.650	88.9 (24.4—95.9)	0.640
≥5	28	26.5 (0.1—164.1)		38.2 (9.0—190)		77.2 (9.8—92.1)		77.8 (36.9—94.2)	
**Haemoglobin (g/dL)**									
<8	17	6.2 (0.1—53.3)	0.519	51.9 (1.4—200)	0.264	24.3 (10.9—80.1)	0.435	63.8 (14.5—95.4)	0.324
≥8	47	25.9 (0.1—160.3)		58.6 (15.4—200)		62.4 (9.7—89.9)		87.3 (35.9—95.4)	
**Leukocyte (cells/μL)**									
<4300	15	0.3 (0.1—17.4)	0.126	26.0 (8.2—169.4)	0.248	17.5 (10.9—74.1)	0.192	63.7 (34.4—91.6)	0.262
≥4300	49	35.5 (0.1—143.6)		110.7 (5.3—200)		64.8 (9.2—91.6)		87.4 (29.6—95.9)	
**Albumin (g/dL)**									
<3,4	34	9.5 (0.1—74.2)	0.189	37.8 (4.2—200)	0.187	37.6 (7.5—87.9)	0.242	64.7 (33.2—95.2)	0.787
≥3,4	30	21.7 (0.2—170.8)		134.9 (16.2—200)		66.21 (12.0—92.9)		88.5 (30.4 –95.4)	

Table S1b. Anti-S-RBD Antibody Level and SVNT (% Inhibition) in ESKD Subjects Vaccinated with mRNA-1273.

Variables	n	Anti S-RBD Antibody Level (AU/mL), median (IQR)				SVNT (% inhibition), median (IQR)			
		pre-vaccination	p-value	post-vaccination	p-value	pre-vaccination	p-value	post-vaccination	p-value
**Duration of HD (years)**									
<5	45	58.2 (0.9—200)	0.383	200 (200—200)	0.529	86.6 (25.8—96.4)	0.217	96.3 (93.0—97.2)	0.767
≥5	16	34.8 (9.9—99.5)		200 (200—200)		64.8 (36.8—88.1)		96.2 (84.0—96.9)	
**Haemoglobin (g/dL)**									
<8	20	132.3 (49.1—200)	0.002*	200 (200—200)	0.293	95.3 (74.0—96.8)	0.001*	96.6 (96.2—97.4)	0.012*
≥8	41	18.9 (0.1—132.8)		200 (200—200)		55.8 (13.7—88.5)		96.1 (84.2—96.7)	
**Leukocyte (cells/μL)**									
<4300	13	119.1 (4.9—193.7)	0.729	200 (200—200)	0.999	59.4 (45.6—94.6)	0.991	96.3 (91.3—97.5)	0.941
≥4300	48	49.6 (1.0—194.3)		200 (200—200)		77.9 (24.2—95.4)		96.2 (95.4—96.9)	
**Albumin (g/dL)**									
<3.4	2	134.6 (69.3—200)	0.333	200 (200—200)	0.999	63.9 (30.6—97.2)	0.655	97.4 (97.2—97.7)	0.097
≥3.4	59	48.5 (1.22—187.5)		200 (200—200)		72.4 (29.1—94.4)		96.2 (91.8—96.9)	

Table S1c. Anti-S-RBD Antibody Level and SVNT (% Inhibition) in ESKD Subjects Vaccinated with BNT-162b2.

Variables	n	Anti S-RBD Antibody Level (AU/mL), median (IQR)				SVNT (% inhibition), median (IQR)			
		pre-vaccination	p-value	post-vaccination	p-value	pre-vaccination	p-value	post-vaccination	p-value
**Duration of HD (years)**									
<5	8	66.1 (9.9—132.9)	0.113	200 (200—200)	0.739	82.0 (46.6—92.2)	0.195	97.1 (96.2—97.2)	0.418
≥5	10	142.3 (88.9—200)		200 (200—200)		93.3 (74.6—96.9)		96.4 (91.5—96.7)	
**Haemoglobin (g/dL)**									
<8	3	121.0 (29.5—200)	0.675	200 (200—200)	0.999	92.6 (73.2—96.9)	0.586	97.2 (97.2—97.3)	0.012*
≥8	15	99.6 (29.2—185.0)		200 (200—200)		89.9 (63.5—95.4)		96.4 (94.5—97.1)	
**Leukocyte (cells/µL)**									
<4300	3	121.0 (91.9—200)	0.457	200 (99.4—200)	0.314	92.6 (36.8—96.9)	0.836	97.2 (48.5—97.3)	0.439
≥4300	15	99.6 (29.2—184.9)		200 (200—200)		89.9 (73.2—95.4)		96.4 (95.7—97.1)	
**Albumin (g/dL)**									
<3,4	11	120.9 (82.4—200)	0.381	200 (200—200)	0.497	91.0 (73.2—95.4)	0.910	97.1 (82.4—97.2)	0.706
≥3,4	7	90.9 (16.4—184.9)		200 (200—200)		78.4 (63.4—95.4)		96.4 (96.3—96.6)	

Table S2a. Bivariate and multivariate analysis of factors associated with high anti-S-RBD Antibody Level (≥59.76 AU/mL) pre-vaccination

Variables	Bivariate analysis		Multivariate analysis	
	OR	p-value	Adjusted OR	p-value
Sex (female)	1.7 (0.8—3.3)	0.117	1.4 (0.7—2.9)	0.319
Duration of HD (≥5 years)	1.5 (0.7—2.9)	0.265	1.4 (0.7—3.0)	0.326
History of COVID (yes)	3.1 (1.4—7.2)	0.005*	2.7 (1.2—6.4)	0.020*
Hemoglobin (<8 g/dL)	1.2 (0.6—2.5)	0.605	1.3 (0.6—2.9)	0.479
Leucocyte (<4300 cells/μL)	0.8 (0.3—1.7)	0.498	0.7 (0.3—1.9)	0.591
Albumin (<3.4 g/dL)	1.2 (0.6—2.4)	0.643	1.1 (0.5—2.4)	0.711

Dependent variable: Anti S-RBD Antibody Level ≥59.76 AU/mL

Table S2b. Bivariate and multivariate analysis of factors associated with high SVNT % inhibition (≥60%) pre-vaccination

Variables	Bivariate analysis		Multivariate analysis	
	OR	p-value	Adjusted OR	p-value
Sex (female)	2.3 (1.2—4.6)	0.014*	1.7 (0.8—3.7)	0.132
Duration of HD (≥5 years)	1.3 (0.7—2.6)	0.424	1.4 (0.6—3.2)	0.373
History of COVID (yes)	9.8 (2.8—34.2)	0.000*	8.6 (2.4—30.9)	0.001*
Hemoglobin (<8 g/dL)	1.7 (0.8—3.6)	0.185	2.4 (0.9—5.8)	0.056
Leucocyte (<4300 cells/μL)	0.4 (0.2—0.9)	0.032*	0.3 (0.1—0.9)	0.026*
Albumin (<3.4 g/dL)	0.8 (0.4—1.64)	0.561	0.7 (0.3—1.5)	0.356

Dependent variable: SVNT % Inhibition ≥60%

KEMENTERIAN PENDIDIKAN DAN KEBUDAYAAN
UNIVERSITAS PADJADJARAN
KOMISI ETIK PENELITIAN
RESEARCH ETHICS COMMITTEE
Jl. Prof. Eyckman No. 38 Bandung 40161
Telp. & Fax. 022-2038697 email: etik.unpad@gmail.com, website: kep.unpad.ac.id

No. Reg.: 0721040377

PERSETUJUAN ETIK
ETHICAL APPROVAL

Nomor: 910 /UN6.KEP/EC/2021

Komisi Etik Penelitian Universitas Padjadjaran Bandung, dalam upaya melindungi hak asasi dan kesejahteraan subjek penelitian serta menjamin bahwa penelitian yang menggunakan formulir survei/registrasi/surveilens/ Epidemiologi/Humaniora/Sosial Budaya/Bahan Biologi Tersimpan/Sel Punca dan non klinis lainnya berjalan dengan memperhatikan implikasi etik, hukum, sosial dan non klinis lainnya yang berlaku, telah mengkaji dengan teliti proposal penelitian berjudul:

The Research Ethics Committee Universitas Padjadjaran Bandung, in order to protect the rights and welfare of the research subject, and to guaranty that the research using survey questionnaire/registry/surveillance/ epidemiology/humaniora/social-cultural/archived biological materials/stem cell/other non clinical materials, will carried out according to ethical, legal, social implications and other applicable regulations, has been throughly reviewed the proposal entitled:

"RESPON ANTIBODI SETELAH VAKSINASI COVID-19 PADA DEWASA"

Nama Peneliti Utama<br>*Principal Researcher*	:	Rudi Wisaksana, dr., Sp.PD-KPTI, Ph.D
Pembimbing/Peneliti Lain<br>*Supervisor/Other Researcher*	:	Bachti Alisjahbana, dr, Sp.PD-KPTI, Ph.D<br>Dr. Agnes Rengga Indrati, dr., Sp.PK(K), M.Kes<br>Marita Restie Tiara, dr., Sp.PD
Nama Institusi<br>*Institution*	:	Pusat Riset Pengelolaan dan Pengendalian Penyakit Infeksi<br>Fakultas Kedokteran Universitas Padjadjaran

proposal tersebut dapat disetujui pelaksanaannya.
hereby declare that the proposal is approved.

Ditetapkan di *Issued in* : Bandung
Tanggal *Date* : 17-05-2021

Ketua,
Chairman,

Nur Atik, dr, M.Kes., PhD
NIP. 19811010 200801 1 019

[illegible]

Figure S2. Ethical approval from the institutional review board

KEMENTERIAN RISET, TEKNOLOGI DAN PENDIDIKAN TINGGI
UNIVERSITAS PADJADJARAN
KOMISI ETIK PENELITIAN
RESEARCH ETHICS COMMITTEE
Jl. Prof. Eijkman No. 38 Bandung 40161
Telp. & Fax. 022-2038697, website: kep.unpad.ac.id, email-sekretariat: etik.unpad@gmail.com
email-sistem: kep.unpad@gmail.com atau kepk.fk.unpad@gmail.com

PSP untuk orang dewasa

PERSETUJUAN SETELAH PENJELASAN (PSP)
UNTUK IKUT SERTA DALAM PENELITIAN
(*INFORMED CONSENT*)

Saya telah membaca atau memperoleh penjelasan, sepenuhnya menyadari, mengerti, dan memahami tentang tujuan, manfaat, dan risiko yang mungkin timbul dalam penelitian, serta telah diberi kesempatan untuk bertanya dan telah dijawab dengan memuaskan, juga sewaktu-waktu dapat mengundurkan diri dari keikut sertaannya, maka saya **setuju/tidak setuju**[*)] ikut dalam penelitian ini, yang berjudul:

RESPON ANTIBODI SETELAH VAKSINASI COVID-19 PADA DEWASA

Saya dengan sukarela memilih untuk ikut serta dalam penelitian ini tanpa tekanan/paksaan siapapun. Saya akan diberikan salinan lembar penjelasan dan formulir persetujuan yang telah saya tandatangani untuk arsip saya.
Saya setuju:
Ya/Tidak[*)]

	Tgl.:	Tanda tangan (bila tidak bisa dapat digunakan cap jempol)
Nama Peserta:<br>Usia:<br>Alamat:		
Nama Peneliti:		
Nama Saksi:		

*) coret yang tidak perlu

Figure S3. Informed consent form
